# Editorial: Feeding a sustainable blue revolution: The physiological consequences of novel ingredients on farmed fish

**DOI:** 10.3389/fphys.2022.1092064

**Published:** 2022-11-24

**Authors:** Luisa M. P. Valente, Benjamin Costas, Françoise Medale, Jaume Pérez-Sánchez, Brett Glencross

**Affiliations:** ^1^ CIIMAR/CIMAR, Centro Interdisciplinar de Investigação Marinha e Ambiental, Universidade do Porto, Matosinhos, Portugal; ^2^ ICBAS, Instituto de Ciências Biomédicas Abel Salazar, Universidade do Porto, Porto, Portugal; ^3^ INRAE, University Pau and Pays de l'Adour, UMR Nutrition Metabolism, Aquaculture, Saint Pée sur Nivelle, France; ^4^ Nutrigenomics and Fish Growth Endocrinology Group, Institute of Aquaculture Torre de la Sal, Castellón, Spain; ^5^ Institute of Aquaculture, University of Stirling, Stirling, United Kingdom

**Keywords:** novel feed ingredients, circular economy, micro and macroalge, microbial biomass, functional diets, low trophic ingredients, insects, fatty acids intracellular fate

Increased reliance on the use of alternative ingredients in the formulations of diets for farmed fish has long been a priority to sustain the unparalleled growth of aquaculture. This need which has led the feed industry to explore many alternative protein and oil sources, has also led to many physiological impacts on those species being fed those alternatives. The intensification of aquaculture under a global climate change scenario also contributes as another major challenge to feed the blue revolution, since there is a need to develop diets that enhance fish robustness. Recent research on farmed fish species has resulted in the development of a range of innovative novel ingredients that are able to sustain fish growth performance, improve fish welfare and resilience, and still assure a safe, nutritious and tasty product for human consumption. Since the suitability and sustainability of vegetable ingredients in diets for carnivorous fish has increasingly being questioned, other resources need to be explored and increasingly we need to identify and develop alternatives that do not compete directly with human food supply. Insects and agrofood byproducts have great nutritional value and may contribute towards a circular economy concept. Biotechnology also offers a wide range of possibilities, including microbial biomass and single cell products, but scale-up is still required for many of these initiatives to realize any serious benefit. Micro and macroalge are also receiving attention, not only as sources of macronutrients, but also for their richness in bioactive compounds. However, the potential of each new feed ingredient has to be thoroughly evaluated before their wide acceptance by the feed industry. There is little to gain by simply transferring risk from one ingredient to another, when ideally what the feed sector needs is greater security of supply, quality and on a cost-effective basis.

This Research Topic on “Feeding the blue revolution” represents a Research Topic of 14 original research articles, highlighting the latest findings related to the evaluation of the physiological consequence of novel ingredients on farmed fish. In the following collection of papers, the readers will note that such an evaluation relies on classical methodological approaches, but also on some cutting edge tools associated with the use of omics to unravel the impact of nutritional clues on the physiology of farmed fish species that are emerging.

Growing interest in the use of insect protein since the inclusion of this ingredient in aquafeed was authorized by the European Union (EU) in 2017 presents new opportunities. In the present volume, Basto et al. have explored the impact of partial and total replacement of fish meal by defatted *Tenebrio molitor* larvae in a comprehensive approach focused on European sea bass (*Dicentrarchus labrax*) growth performance and nutrient utilization, but also further addressed the underlying physiological mechanisms involved in nutrient metabolism. In this carnivorous species, the authors concluded it was feasible to replace up to 80% fish meal without growth impairment, and still assuring fillet nutritional value for consumers. Another important insect species, black soldier fly, *Hermetia illucens*, was assessed by Li et al. as protein source for tongue sole (*Cynoglossus semilaevis*). The replacement of 25% of the fish meal by defatted black soldier fly larvae meal had positive effects on growth, but with some drawback effects on feed conversion. They also noticed intestinal structural damage and abnormal liver function at higher replacement levels. These studies highlighted the potential of insect meals to be included in aquafeeds, but identify that there are maximal inclusion limits depending on the fish species considered. It is also clear that further studies are warranted to fully validate such nutritional approaches at farm scale considering the full production cycle.

Kousoulaki et al. considered a series of low trophic ingredients (*Schizochytrium limacinum*, the diatom *Phaeodactylum tricornutum*, *Hermetia illucens* and tunicate meal from *Ciona intestinalis*) in fishmeal and fish oil free diets when fed to Atlantic salmon (*Salmo salar*). Extensive raw material and dietary chemical characterisation was undertaken to identify constraints and opportunities for using such novel ingredients. Overall, each of the studied ingredients were well accepted by the fish, resulting in high performance and efficiency. It was highlighted that the relevance and impact of the quality of fishmeal and fish oil on comparative studies with such novel ingredients is an important consideration.

Insights in fish feeds for increased circularity and resource utilization were provided by Naya-Català et al. who evaluated processed animal proteins (PAPs), insect meal, yeast, and microbial biomasses in diets for gilthead sea bream (*Sparus aurata*) juveniles. This study focused on the interaction between intestinal microbiota and host transcriptomics to aid the identification of fish diets promoting gut health and metabolic homeostasis, and ultimately, the overall health of farmed fish. Using a circular economy concept, Terova et al. assessed crude glycerol, a primary by-product of biodiesel production, for heterotrophic cultivation of *Schizochytrium limacinum*. The resulting biomass, a rich source of docosahexaenoic acid, was further shown to be a useful alternative to marine-derived raw materials for European sea bass feeds, providing a much needed additional source of essential omega-3 fatty acids for indirect human consumption. However, the feed cost-effectiveness for that resource still needs to be improved.

Algae were examined in several papers in this Research Topic. García-Márquez et al. demonstrated the capacity of the microalga *Chlorella fusca* (15% inclusion level) to promote growth, metabolism and digestive functionality in a low trophic omnivorous fish species, the thick-lipped grey mullet (*Chelon labrosus*). In another paper, Fang et al., showed that a newly isolated strain of Haematococcus pluvialis GXU-A23 improved the growth performance, antioxidant and anti-inflammatory status, metabolic capacity and mid-intestine morphology of juvenile *Litopenaeus vannamei*. A 50 g/kg *Haematococcus pluvialis* was proposed to be used as a green additive in aquafeeds due to its rich astaxanthin and polyunsaturated fatty acid content. Ferreira et al. also explored a range of natural highly nutritious alternatives to replace fishmeal in gilthead sea bream diets. They found that microalgae were a good source of protein and lipids, able to promote the accumulation of LC-PUFAs in muscle, whilst selenised yeast was a good vehicle to fortify selenium levels in fish. However, the use of *Laminaria digitata* as strategy to increase iodine muscle content still requires further evaluation.

Functional diets continue to develop as strategies to boost fish health status. Hydrolyzed *Debaryomyces hansenii* yeasts were shown to modulate physiological responses in plasma and immune organs of Atlantic salmon (Morales-Lange et al.) counteracting possible consequences of hypoxic stress. Balbuena-Pecino et al. used an *in vivo* and *in vitro* approach to evaluate hydroxytyrosol-rich extract from olive juice (major phenolic compounds found in olives) as an additive in gilthead sea bream juveniles fed a high-fat diet. That study highlighted the beneficial use of hydroxytyrosol as a way to improve fish muscle-skeletal condition. The metabolic effects of flavonoids included in diets for hybrid grouper (*Epinephelus fuscoguttatus♀×Epinephelus polyphekadion*♂) was evaluated by Luo et al. Classical feeding trials combined with extensive tissue metabolomics analysis, helped to shed light on metabolic adaptations induced by such functional feeds. The use of glycerol, a by-product of biodiesel, was also evaluated in diets for rainbow trout (*Oncorhynchus mykiss*) and European sea bass. Viegas et al. suggested that these fish are able to catabolize glycerol and incorporate it into carbohydrates, but species metabolic differences were identified. An additional study by the same authors recommended a dietary glycerol supplementation of up to 2.5%, as it had no major effects on fish lipid metabolism or fat accumulation and therefore had some potential as a useful energy source (Viegas et al.). Finally, Selvam et al. provided new insights into intracellular trafficking of fatty acids using a rainbow trout intestinal epithelial cell line (RTgutGC) as an *in vitro* model. This model provided comparable results to similar mammalian cell lines as well as *in vivo* studies in fish or mammals. It was concluded that carbon chain length and saturation level of fatty acids differently regulate their intracellular fate during fatty acid absorption.

Overall, the “Research Topic” highlights a variety of current research in the area. This set of studies also shows how classical and novel research strategies are increasingly being used in tandem to underpin future directions to increase the range of alternative ingredient options for use in feeding farmed fish species. Beyond the theoretical aspects of these studies, increasing use of novel research strategies is providing an objective understanding of the physiological responses by fish to a growing range of alternative ingredients.

